# Radiological and histological findings in ancient salt mummies from the salt mine of Douzlākh, Iran

**DOI:** 10.1371/journal.pone.0250745

**Published:** 2021-04-30

**Authors:** Lena Maria Öhrström, Herman Marquez, Roger Seiler, Beata Bode, Abolfazl Aali, Thomas Stöllner, Frank Jakobus Rühli

**Affiliations:** 1 Faculty of Medicine, Swiss Mummy Project, Institute of Evolutionary Medicine, University of Zurich, Zurich, Switzerland; 2 Institute of Pathology and Molecular Pathology, University Hospital Zurich, Zurich, Switzerland; 3 Archaeological Museum of Zanjān, Emaarate Zolfaghari, Zanjān, Iran; 4 Ruhr Universität Bochum, Institut für Archäologische Wissenschaften, Bochum, Germany; 5 Deutsches Bergbau-Museum Bochum, Research Department & Mining Archaeology Research Branch, Bochum, Germany; University of Florence, ITALY

## Abstract

Computed tomography studies and histological analyses were performed on the mummified remains found in the Chehrābād salt mine in northwestern Iran. The ancient salt mummies are dated to the Achaemenid (550–330 BC) and Sassanid (3rd–7th century AD) time period and died in mining incidents. The aim of the study was to describe the radiological and histological findings of several ancient Iranian salt mummies with special interest in pathological and postmortem changes. The mummified remains show multiple traumatic alterations, such as fractures and signs of massive compression. Histological analyses can clearly differentiate soft tissue, however the preservation status is variable. These Iranian salt mummies are a rare example of the ancient Iranian population. The soft tissue and organs are well preserved, however in different degrees due to the varying conditions.

## Introduction

Several ancient Iranian salt mummies and mummy-parts have been found in the Chehrābād salt mine in northwestern Iran between 1993 and 2010. C14-dating revealed that they are either dated to the Achaemenid (6^th^– 4^th^ century BC) or the Parthian-Sassanian (2^nd^– 6^th^ century AD) time period [1–4]. In contrast to artificially embalmed mummies known from different cultures, the Iranian Saltmen have been naturally mummified [5]. Different ways of natural mummification may occur depending on climatic and environmental conditions. In the case of the salt mummies the mummification process was induced by salt. As in the artificial mummification procedures in ancient Egypt, the preservation of soft tissue was mainly caused by the hygroscopic effect of the salt [5]. The resulting dehydration inhibits bacterial growth and arrests decomposition.

The Chehrābād Saltmen (SM) are of special archeological and historic interest. They are rare examples of individuals dating to the ancient Persian periods and are, to date, the only known preserved salt mummies worldwide [6].

### The Archaeology of the Iranian Saltmen

**Fig 1:**
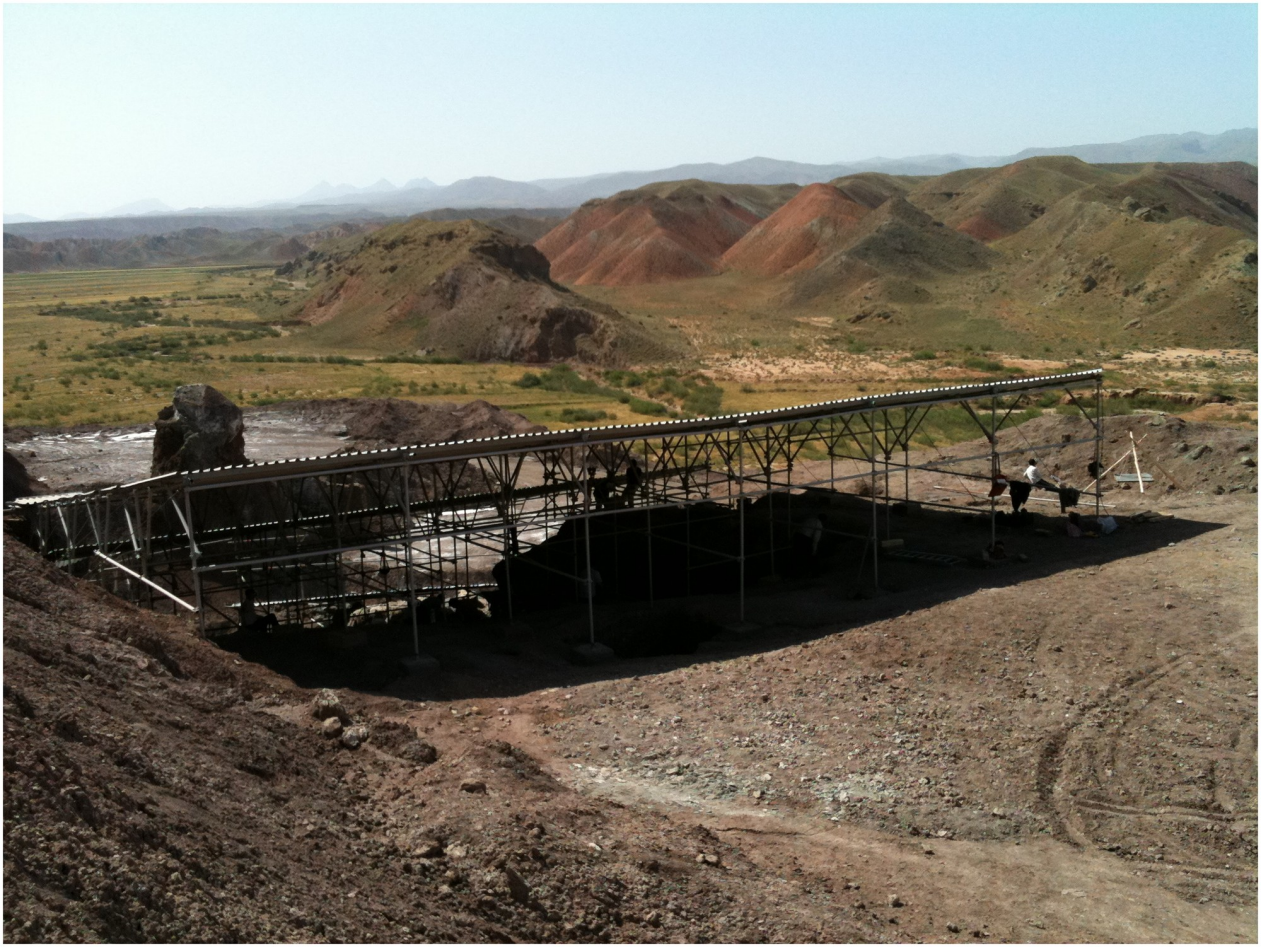
Chehrābād salt mine; excavation site. Photo taken by Frank Rühli.

The Chehrābād salt mine of Douzlākh is located 75km northwest of Zanjan city near the village of Hamzelou in northwestern Iran. The mine is part of a large salt dome with an area of ca. 20 hectare; 1350 meters above sea level, latitude N 36°54’52’’, longitude E 47°51’25’’ (Fig 1). The rock includes red, green, and grey marl and clay deposits with gypsum, plaster and salt fragments [4], pp. 17–18.

Mining at Chehrābād is an exceptional example of a salt mine that has been exploited during several time periods. Due to the long-running mechanical excavation of salt, deposits of this mineral have emerged from the underlying layers. Still active until recently, this salt deposit was considered important in recent years, but also in ancient times. Evidence of salt exploitation has been reported during four extended periods, the Achaemenid (6^th^– 4^th^ century BC) and Sassanid (3^rd^– 7^th^ century AD) eras as well as the middle and late Islamic periods (11th/12th. Century AD and 18th–20th century AD, respectively).

To date, the human remains of at least eight different individuals have been found at this location and their descriptions are detailed in Aali et al [2].

When the first salt mummy was discovered, the salt mine was still active. Upon the discovery of the remains of Salt Man #1 (SM1) in the winter of 1993, field work was conducted in the same year. At this location, salt extraction continued through 2004 until the remains of SM2 and SM3 were accidentally discovered. In the meantime, mining activities have ceased and the site has been protected by Iranian Heritage laws since 2009. Since the discovery of human remains as well as multiple artifacts, archaeological field work at the Chehrābād salt mine has resumed.

With the support of the German Research Foundation (DFG), systematic excavations were conducted in 2010–2017 by a multidisciplinary research team, led by the Iranian Cultural Heritage Centre Zanjān (Miras Farhangi Zanjān; Ruhr University of Bochum, Institute for Archaeological Studies and the Deutsches Bergbau-Museum Bochum as the main project partner, other collaborators from Tehran, Zurich, Oxford, Vienna, Paris and Besançon) [4,7]. This field and laboratory work discovered SM6 and assured the archaeological and stratigraphical contextualization of the site. Several organic accessories such as fur and textile clothing as well as working tools were excavated and display the rich material background of the ancient salt mining communities [4,8,9].

The archaeological excavations have yielded exciting results on the technology and logistics of the old mining processes [4] and there is evidence of at least three mining accidents.

Most of the Saltmen were likely miners and died in the mining accidents.

## Material and methods

### Material

**Fig 2:**
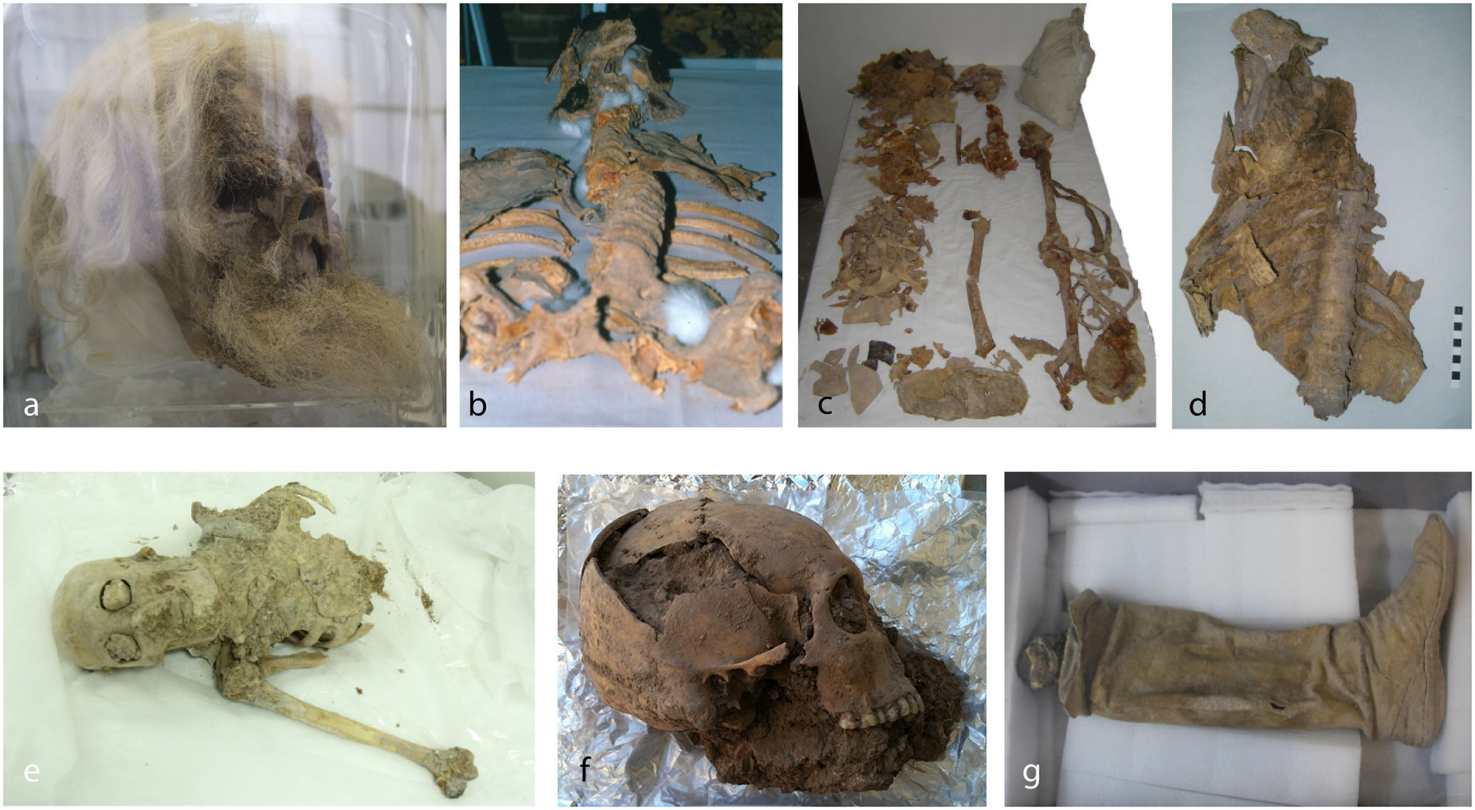
a-g: Photos of the salt mummies; a) SM1; b) SM2; c) SM3; d); thorax found nearby of SM1; e) SM5; f) SM6; g) leather shoe with lower leg, likely belonging to SM1, SM7 or SMX. Photos taken by Frank Rühli (e, f, g), Abolfazl Aali (b, c) and Lena Öhrström (a, d).

Hitherto human remains of at least eight individuals have been found, dating either to the Achaemenid or Parthian-Sassanian time period (Fig 2).

SM1 was found during mining activities in 1993. A mummified head with long hair and a beard is preserved with a gold earring in his left ear (Fig 2a). C-14 dating indicates that the miner lived in the early Sassanian period (220–390 AD) [1,2]. In the subsequent rescue excavation, a left lower leg inside a well-preserved leather boot was found nearby, eventually belonging to the same individual (Fig 2g). Further mummified remains including cervical vertebrae and a second maxilla were discovered in the same area and delivered to the excavators A.A. Mir Fattah and H. Sobouti and then to the National Museum of Iran. It was determined from these remains that there must be at least two individuals. Since these investigations took place at a later date, the mummy is named SM7. Additional mummified remains also in context with excavations around SM1, but with unclear circumstances of how these were found, are stored in the archive of the National Museum of Iran in Tehran, including part of a thorax, a humerus, and an ulna as well as some loose ribs (Fig 2d). These remains also belong to SM1, SM7, or another individual. Molecular investigations to clarify any relationships are ongoing. SM2 was found during mining activities in 2004 (see above). He is mostly skeletonized and highly fragmentary; however, some soft tissue, including hair and a beard, are preserved (Fig 2b). He is dated to the later Sassanid time period (430–570 AD). SM3 was also discovered during mining activities in 2004. He was found under a large salt block and was heavily compressed and mostly skeletonized. Fragmentary bones with some soft tissue remnants are preserved (Fig 2c). The remains were dated to the Achaemenid time period (410–385 BC) [1,2]. Further studies showed that these human remains probably belong to two different people. Regardless, these remains were designated as SM8 [10].

No complete radiological analyses have been performed of SM2 or SM3, since they are mostly fragmentary and skeletonized. SM4 (SM4) was found during the first excavation season in 2004/5 and is the best preserved mummy. He was an adolescent of about 15 years and dated to the Achaemenid time period [1,2,11]. The radiological findings of this individual have been described separately [9]. SM5 was found during the second field season in 2005. The body had been laying under a large rock, which was removed to excavate the mummy. SM5 is largely skeletonized and the bones are almost fully preserved (Fig 2e). The head shows skin and hair remnants and soft tissue is found on the arms, legs, and thorax. Additionally, a few fragements of clothes are also preserved. Radiocarbon dating [2,4] assured the assignment to Achaemenid time period (ETH (41106: 95.4% probability: 730BC (6.1%) 690BC; 550BC (89.3%) 380BC)). Remains of SM6 consists of a cranium with multiple fractures (Fig 2f) and likely part of a pelvis that was found nearby. He was excavated in 2010 [2,4]. The head was dated to the Sassanid period (430–620 AD, ETH-41107).

While SM1 is on exhibit in the National Museum of Iran in Tehran, the other Saltmen are currently exhibited in the Archaeological Museum of Zanjān, Zanjān, Iran.

### Methods

Radiological analyses using X-ray were previously performed on SM1 and SM4 [3,11]. These investigations are not discussed here. Due to the availability of improved imaging technologies, the radiological analyses were repeated with Computed Tomography (CT).

New radiological investigations were performed at the Tehran Heart Center, on a clinical Siemens Somatom Definition Flash CT-Scanner. Imaging parameters were as following; 512x512 matrix size, between 0,49 and 0,89um Pixel spacing, between 107 and 636 mA tube current, between 0,6 and 1mm slice thickness and between 80 and 140 kv tube voltage. Radiological analyses of SM4 and SM6 were performed separately in 2011 at the Pardis Nor Medical Center in Tehran on a clinical CT scanner (Siemens Sensation 16) with the following imaging parameters (for SM6): 5mm slice thickness, 120kvP, 308mA tube current, 512x512 matrix size, and 0,39um pixel spacing.

The datasets were processed with DICOM reading software (OsiriX MD v.8.0.1, Pixmeo, Switzerland), including multi-planar reconstructions (MPR) and three-dimensional volume rendering (3D VR).

Histological analyses were performed at the Institute of Pathology and Molecular Pathology of the University Hospital, Zurich, Switzerland. The tissue samples were rehydrated and fixed by immersion in buffered 4% formalin. Specimens containing bone were decalcified in EDTA. All samples were embedded in paraffin. 2 μm thick sections were cut and all were stained with hematoxylin and eosin (HE), elastica Van Gieson (EVG), PAS, Grocott and Gram stain according to routine standard procedures. An overview of the samples is summarized in Table 1.

**Table 1:** Overview of the histology samples.

Salt mummy	Sample region	Tissue type	Bacterias	Fungi	Sample number
1	throat	collagen rich tissue (fascia?)/fatty tissue	a lot	probably	112
1	throat	thick collagen bundles/fatty tissue (tendon?/fascia?)	no	a lot	106
1	throat	collagen rich tissue/refractile materials (cristals)	a lot	few	107
1	hair	hair/plant particles	no	no	104–2
1?	left rib	bone and periostal soft tissue	little	no	111
3	right tibia	cortical bone	no	no	124
4	right arm pit	skeletal muscle and fatty tissue	no	no	115
4	pubic symphysis	cartilage and soft tissue	some	no	116
4	left pectoral muscle	skelettal muscle/fatty tissue	no	no	117
4	left clavicle	spongiosa bone	no	no	118
5	right femur	cortical bone	no	no	121
6	right parietal bone	bone (spongiosa and cortical)	no	no	123
unclear	isolated maxilla	bone	no	a few	102
unclear	inner thorax	unclear, only collagen fibers	not tested	not tested	3303
unclear	vertebral/spinal	unclear, only collagen fibers	not tested	not tested	3305

Age estimation was performed by using the bone age status of the hand, feet and knee, the dental status, as well as physical characteristics. Standard sex estimation in skeletal material is based on the analyses of the pelvis bones [12]. While additional features, such as skull shape, are also utilized additionally to standard sex determination methods, these features were found to be too indeterminate to be used on their own [13]. Molecular sexing using ancient DNA (aDNA) was applied for SM5.

Ethics statement: No permits by ethics committee were required for the described study, since the investigated ancient human remains are older than 1500 years and no living relatives are known. The investigations were done according to our code of ethics:

IEM Code of Ethics http://www.iem.uzh.ch/en/institute/iemcodeofethics.html.

The research institute of the cultural Heritage and Tourism of Iran (RICHT) granted permission for the field research.

## Results and discussion

All individuals show multiple traumatic lesions and postmortem alterations, including fractures and compression-related lesions as well as shrunken tissue and organs. The traumatic injuries are most likely the results of the various assumed collapses that occurred in the ancient salt mines. Similar fractures are found in modern victims of high velocity trauma or mining accidents [14].

Histologically, various tissues, such as connective collagen fibers, adipocytes of the fatty tissue, vascular tissue, and hair structure are well identifiable. However, bone tissue is best preserved. Several samples show a high bacterial load and presence of fungi, indicating colonization, that most likely happened postmortem.

### Salt Man #1 (SM1)

#### Radiological findings

**Fig 3:**
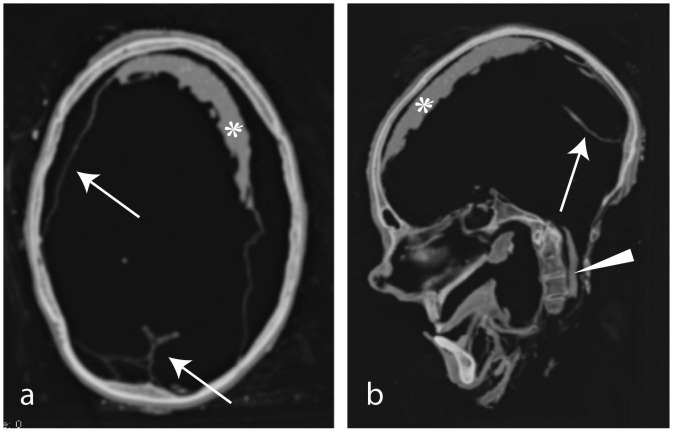
a) axial slice and b) MPR reconstructions of the head of SM1. Stars: Shrunken brain remnants.; arrows: Dura remnants; arrowhead: Spinal nerves and dura remnants. C: Dislocated clivus.

The partially mummified head including the proximal cervical spine (C1 to C3, and upper portion of C4) is preserved (Fig 3). The alignment is not intact, as the vertebral bodies are moved to the side and tilted frontally. A distal fragment of the left clavicle bone is preserved, together with the acromion from the left scapula, although both fragments are dislocated. The mummy wears a circular, metallic earring in the left ear. Hair and a beard are also preserved. The frontal and maxillary sinus, as well as the ethmoidal cells, together with the mastoid, are well-pneumatized. The cranial plates show no fractures. The ethmoidal cells are fractured at several locations. The ramus of the right mandible is fractured between the condylar process and the coronoid process, and also at its distal portion. The right maxillary bone and the lateral wall of the orbit show multiple fractures. Only small portions of the orbits remain. No anatomical structures can be identified within the orbits. Some dehydrated brain remnants are found fronto-parietally and parts of dura, falx, and tentorium are preserved. Some remains of the spinal cord/spinal dura are observed in the spinal canal (Fig 3b).

#### Dental status

While all teeth in the lower jaw are present, some of the molars in the upper jaw are missing, the remaining root of 17 (after the FDI nomenclature) shows apical translucency, ca. 4mm in diameter. Premolars and molars show extensive dental wear, i.e. the occlusal surface is completely worn to the dentine, and in the case of 46 dental wear reaches even the dental pulp. The other teeth have slight to moderate wear. There is an overall increase in the distance between the cementoenamel junction and the alveolar crest, partially due to the loss of dental hard tissue, but in the molar region of the lower jaw clearly because of chronic periodontitis. Postmortem fracture only occurs in the crown of 25. In conclusion, although there are no signs of caries, the molars and premolars are heavily compromised by dental wear and periodontitis.

**Fig 4:**
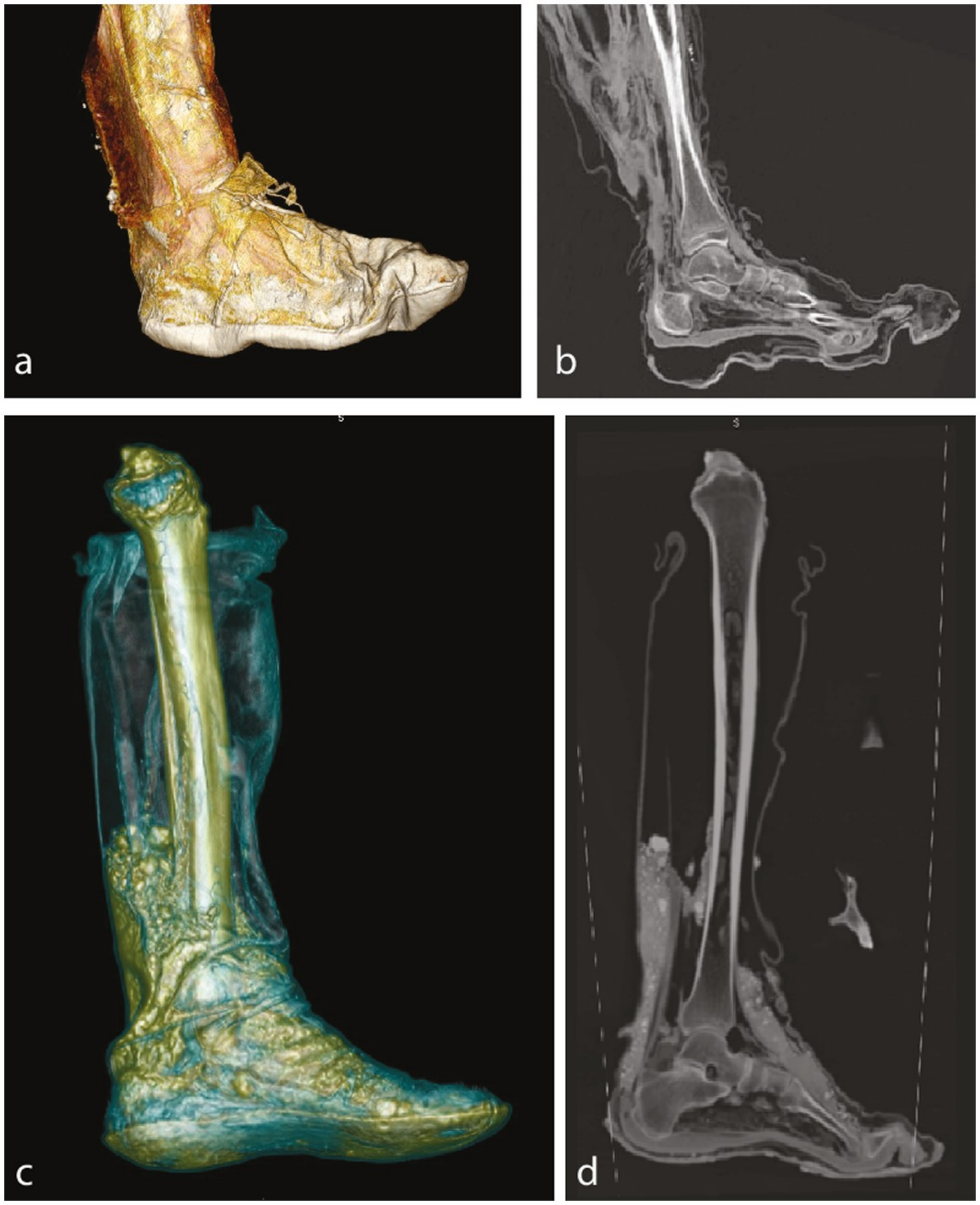
Comparison of shoes for SM1 vs SM4. a) 3D volume rendering of the shoe of SM4, b) sagittal MPR, c) 3D volume rendering of the lower leg of the assumed SM1, d) corresponding MPR. Note that no socks are worn.

Found in close proximity of SM1’s head was a partially mummified lower leg, including the foot in a well-conserved leather boot. The foot, including the ankle, are fully mummified, whereas the proximal lower leg is skeletonized. The knee joint is missing. No osseous pathologies are observed. The epiphyseal plates are closed, indicating an adult age. The goat-leather boot is very well preserved and partially filled with soil deposits, such as sand and probably some stones. No socks were worn. The toes are in a flexed position with almost no space in front of the toes, indicating the fit of the shoe was possibly a bit too short. The boot style is different than that worn by SM4; SM1 wore a tall shaft leather boot, while the shoes of SM4 are ankle-high with a sole (Fig 4).

As described above, further contextual remains may belong to SM1 or possibly other additional individuals (e.g. SM7) [10]. These remains include part of a thorax with partially preserved vertebral column, a fragmented right scapula and shoulder, with preserved soft tissue but no inner organs, humeral and ulnal bones as well as some loose fragmented ribs. Additionally, three thoracic vertebrae with some dura remnants and a part of a maxillary bone with teeth and very little soft tissue are preserved. The vertebrae and thorax may belong to SM1, but the maxillary bone does not, as the skull has two intact maxillary bones. Instead, this fragment likely belongs to another individual interred in the vicinity. The CT scan showed closed epiphyseal plates, indication an adult age, but no substantial degenerative changes. Some peri/postmortem fractures are present, but no pathologies are evident. Thus far, these skeletal remains have not been radiocarbon dated.

#### Histological findings

**Fig 5:**
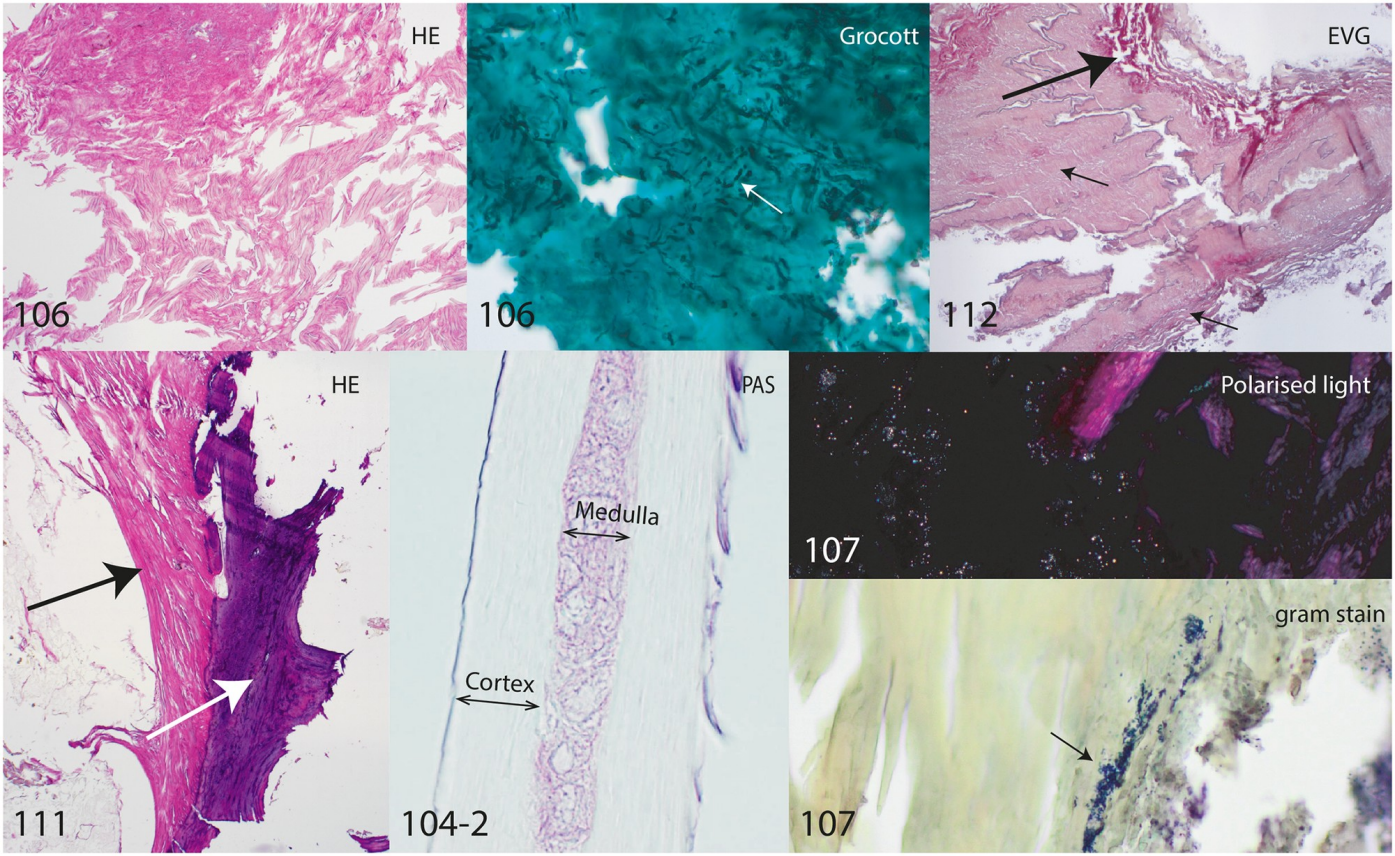
Histology samples of SM1. Sample numbers 106 & 112: Throat area. Note the well-preserved collagen fibers on the HE stain of sample 106. The Grocott stain shows multiple fungi (white arrow). 112: Note the collagen rich perivascular tissue (big arrow) and part of the tunica muscularis of an artery (big arrow). 104–2: Hair. 107: Throat area, the gram stain indicates a high amount of gram positive bacteria (arrow). The polarized light stain indicates crystalline birefringent substance mixed with the tissue. 111: Part of left rib, probably belonging to SM1 with bone (white arrow) and periostal soft tissue (black arrow) with collagen fibers and fatty tissue. Histological staining HE: Hematoxylin and eosin. EVG: Elastica Van Gieson PAS: Periodic acid-Schiff. Grocott: Silver stain for detection of fungi. Gram stain: For detection of bacteria.

Several samples have been analyzed, including soft tissue from the neck and throat, as well as hair and ribs. Fatty tissue including blood vessels as well as collagen bundles could be identified in the throat region. Bone and periosteal soft tissue are visible and the hair structure is still intact. An abundance of fungi and bacteria are identified particularly in the throat region (Fig 5).

**Fig 6:**
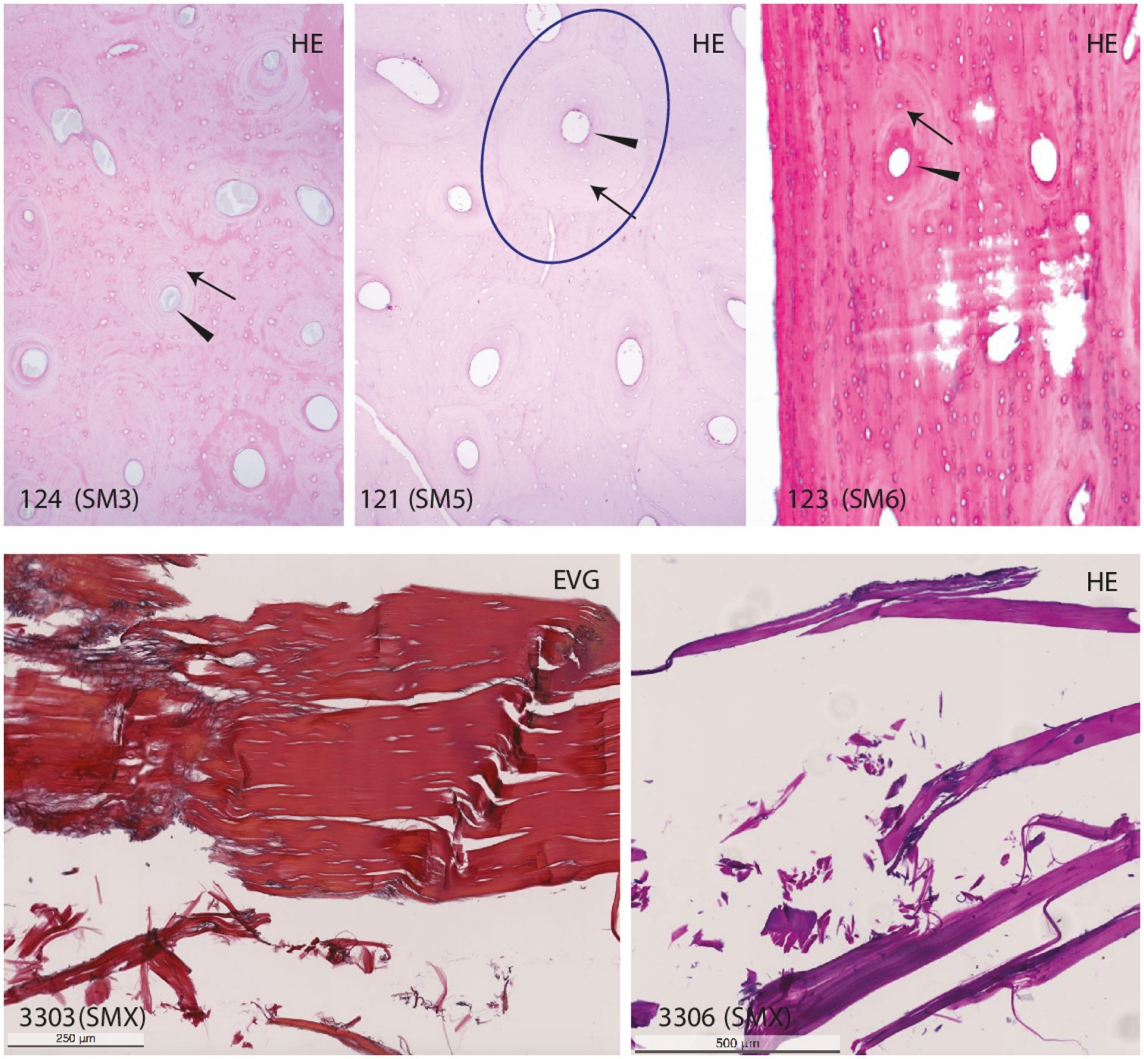
Histology samples of SM3, SM5, SM6 and SMX. Bone samples from SM3, SM5 and SM6 showing normal necrotic cortical bone matrix. Note the intact osteon structure (circle). Arrowhead: Haversian canal with remnants of blood vessels. Arrow: Osteocyte lacuna. Samples 3305 and 3305 from the inner thorax of SMX, with unclear excavation context, show collagen fibers without assignable tissue. Histological staining HE: Hematoxylin and eosin stain, EVG: Elastica Van Gieson.

Histological analyses of the thorax found contextually separate only identified collagen fibers. No tissue structure could be identified, probably due to a poor state of preservation (Fig 6; 3303 and 3305).

### Salt Man #2 (SM2)

No radiological analyses were performed on SM2. For the histological analyses, sample size was too low.

#### Dental status

The following findings can be obtained from the available photos: Dental wear lays the dentine free in patches on the occlusal surface of 15 and 16. There are multiple postmortem fractures present, (15, 13, 11, 21) and on the left side, teeth not preserved from 26 onwards. The periodontal status is normal and no carious lesions are visible.

### Salt Man #3 (SM3)

No radiological analyses have been performed on SM3 because the remains are mostly skeletonized and highly fragmented.

#### Histological findings

A sample from the right tibia shows regularly structured mineral matrix of normal ‘necrotic’ cortical bone (Fig 6; 124). No bacteria or fungi are identified.

### Salt Man #4 (SM4)

Radiological findings are discussed elsewhere, as mentioned above.

#### Histological findings

**Fig 7:**
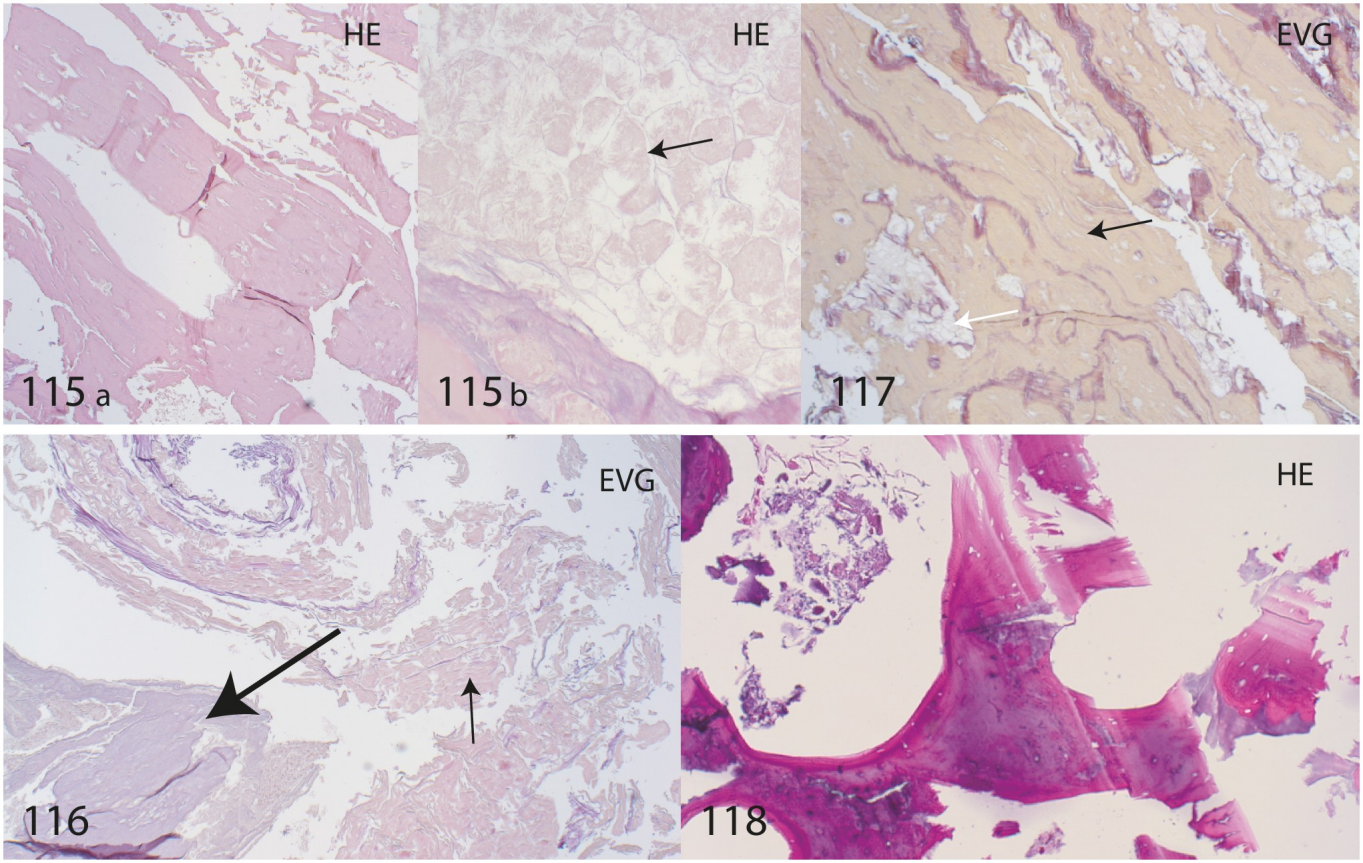
Histology samples of SM4. Sample number 115: arm pit with skeletal muscle (a) and fatty tissue (b) with adipocytes (arrow). 116: os pubis with cartilage tissue (big arrow) and surrounding soft tissue with connective tissue (small arrow). 117: Pectoral muscle with skeletal muscle fibres (black arrow) and fatty vacuoles (white arrow). 118: Clavicle bone with normal ‘necrotic’ spongiosa. Histological staining HE: Hematoxylin and eosin. EVG: Elastica Van Gieson.

Several samples of SM4 have been investigated, including soft tissue from the armpit and left pectoral muscle as well as a bone sample from the clavicle and tissue from the os pubis region. Skeletal muscle, fatty tissue, spongiosa and cartilage can be visualized. No fungal structures were seen, however some bacteria were identified in the pubis region (Fig 7).

### Salt Man #5 (SM5)

The body of SM5 is almost fully preserved, however, it is mainly skeletonized and divided into several parts. Missing parts of the skeleton include the lower thoracic vertebrae (T6 to T11), the left scapula, the proximal portion of the humerus, and the right hip bone.

#### Radiological findings

The body shows multiple traumatic alterations, such as fractures and signs of extreme compression. No intravitam pathological alterations were found on the bones. The epiphyses of the hand, as well as the long bones are closed, indicating an adult age estimation. According to the parameters proposed by Walker and Lovejoy [15], radiological examination of the clavicle estimates the age range of this individual to be approximately 45–49 years old. However, dental eruption patterns suggests a younger individual, which will be discussed later.

**Fig 8:**
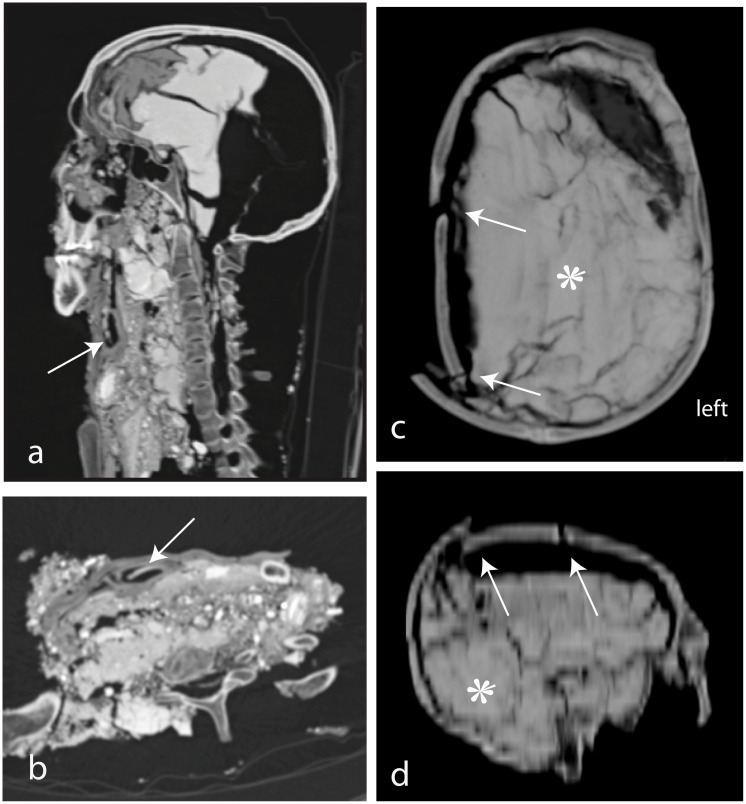
(SM5 & SM6). a) sagittal and b) axial MPR of SM5 showing the probable trachea remnants (arrows). c) axial view and d) coronal MPR of the cranium of SM6. Arrows indicating fractures and disrupted sutures. Star: Soil.

The cranium shows no fractures. The clivus is dislocated and moved to a caudal position, however the sella turcica remains intact. The maxillary, sphenoidal, and frontal sinuses and mastoids are well-pneumatized. The cranial cavity shows some brain remnants in the frontal region and parts of the dura. However, the cavity is largely filled with an isodense and compact structure, probably soil or salt and small stones (Fig 8a & 8b). The orbits show no fractures, and are mainly filled with soil. The orbit contents are not preserved.

The thorax is heavily compressed, mainly on the left side, and the ribs have multiple fractures on both sides. On the right side, seven proximal ribs are preserved and anatomically positioned; on the left side, five proximal ribs are preserved. Parts of the mediastinum can be seen, but no thoracic organs can be clearly identified. Remnants of a hollow organ are observed close to the beginning from the pharynx, dividing into two parts at the distal end, likely corresponding to the trachea (Fig 8a & 8b).

All cervical vertebrae and thoracic vertebrae T1 to T5 are preserved and anatomically positioned. No fractures or degenerative changes are present in these vertebrae. The dens axis is well-positioned, although the whole spine is slightly rotated to the right from T2 onwards. Vertebrae T6 to T11 are missing. Lumbar vertebra, including T12 as well as the sacral bones, are preserved. A slight decrease in the height of vertebral bodies L4 and L5 is noted as well as fractures of some spinal processes. The sacrum is dislocated at S3-S4.

**Fig 9:**
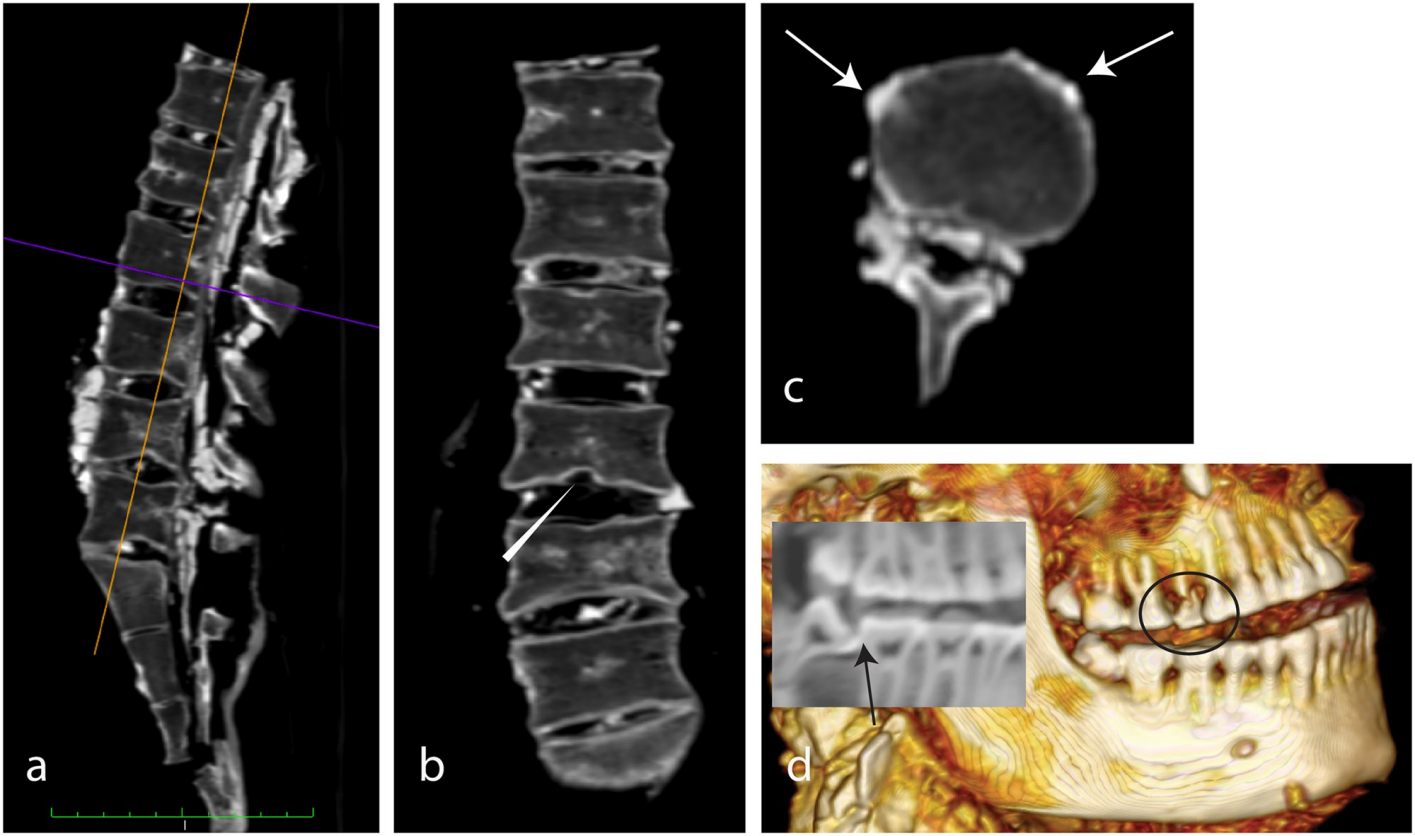
SM5. a) sagittal MPR of the vertebral column, b) coronal MPR, c) axial slice showing degenerative changes with osteophyte formation (arrow) and Schmorl’s node (arrowhead). d) 3D Volume rendering of the right side of the dentition (circle: Fractured 15) with sagittal 3D multiplanar reconstruction (insert). Arrow: Carious lesion on occlusal surface of tooth 48.

Several osteophyte formations and irregularities of the upper and bottom plates are found (Fig 9a–9c). Some remnants of the spinal nerves are seen in the spinal canal in addition to some soil.

Both clavicles and the sternum are intact and the sternoclavicular joints are preserved. The right shoulder is preserved including the proximal part of the scapula and the entire humerus. There is a small fracture at the inferior angle of the scapula. The right elbow is completely dislocated and not in an anatomical position. However, the right ulnar, radial bones, and the hand are all preserved and intact. The left shoulder as well as the left scapula and proximal humerus are missing. The distal part of the humeral bone and the forearm are preserved with the elbow joint intact. Part of the distal radial bone is missing. The left hand is preserved, but the 5th metacarpal/phalanges are dislocated. No fractures are present.

Part of the left hip bone (the ilium) and part of the fractured coccygeal bone are preserved. The ischium and pubis are missing. The right hip joint, including part of the acetabulum, is partially preserved.

The right upper and lower limbs including the talus and calcaneus are preserved and anatomically positioned. The knee is flexed at approximately a 30° angle. Tarsal, metatarsals, and phalanges are also preserved, but separated from the ankle joint. The right femoral neck shows multiple fractures.

#### Dental status

All teeth are present and only slightly worn (except the first molars, where the dentin is largely exposed. The third molars show no dental wear at all. The only carious lesion is found on the occlusal surface of 48 and the bone loss in only low. So, the dentition is in good condition. The crown of the tooth 15 is fractured postmortem (Fig 9d).

#### Histological findings

A sample from the right femur shows normal ‘necrotic’ cortical bone without pathologic changes. No bacteria or fungi are identified (Fig 6; 121).

### Salt Man #6 (SM6)

A fragmented skeletonized cranium and part of the pelvis have been found of SM6.

#### Radiological findings

The mandible and the part of the right parietal bone are missing; the cranial cavity is filled with soil (Fig 8c & 8d). The skull shows multiple traumatic alterations (which occurred peri-as well as porstmortem), including several fractures as well as ruptured sutures, partially leading to impression of the cranial plates. The coronal suture as well as the frontal portion of the sagittal suture are blown apart and the zygomatic process is disrupted at the zygomatico-maxillaris suture on the right side. Additionally, the sphenosquamosa suture, frontozygomatica suture, and sphenofrontalis suture are slightly disruptured on the right side. The left parietal is fractured in the middle portion. The right orbit is fractured at the frontal process of the maxillary bone. The right temporal bone, frontal bone, and the right orbit are also fractured.

#### Dental status

The dental status of SM6 cannot be assessed radiologically, mainly due to the poor resolution of the MPR. The following findings can be obtained from the available photos: postmortem loss of 17, slight dental wear, and small cervical caries on the exposed tooth necks of 16 or 21. Several rows of light linear enamel hypoplasias on the buccal surfaces of the anterior teeth are visible.

#### Histological findings

Spongiosa and cortical bone can be visualized on a sample from the parietal bone. No fungi or bacteria are identified (Fig 6; 123).

### Age and sex determination

All Saltmen are assumed to be male, however not all the remains could be sexed thus far. External features such as facial hair are present in several mummies (e.g. SM1 and SM2), which confirms such individuals as male. The sex of SM3 has been estimated as presumable male by some of the skeletal remains [10]. SM4 is estimated as being male. Molecular sexing using ancient DNA (aDNA) was used for sex determination of SM5, confirming a male sex. The biological sex of SM6 is not fully identified. Only the imprecise data from the skull shape estimated this idividual as a possible male. In this case, aDNA would clarify the sex, however these analyses are still ongoing.

Age determination in adult skeletal material remains rather difficult [16]. In subadult individuals, it is usually performed by estimating bone age of the hand, according to the hand atlas of Greulich and Pyle [17]. Additionally, bone atlases of the knee and feet can also be used [18,19]. In forensic medicine, age estimation includes physical examination, assessing dental eruption patterns, and bone age determination [20]. In adults, the sterno-clavicular joint and the ossification status of the medial part of the clavicula is frequently used for age assessment [15,21]. Other methods to determine age utilizing criteria such as prevalence of caries, dental wear, or alveolar bone loss are problematic, at least from the age of 14 onwards, when dentition is fully developed [22], and when only a few individuals of a population are assessed [23]. Food preparation and consumption are often not fully understood in ancient populations, but have a crucial influence on the development of oral diseases [24]. Therefore, when relaying upon dentition, only a rough estimation of the age at death is possible.

With only the head preserved, age determination for SM1 is approximate, at best. The missing teeth, probably due to periodontitis, and the heavy dental wear estimate this individual was likely an older adult. SM4 is of juvenile age, estimated to be 15–16 years old [9]. The radiological analyses of SM5 indicate an age range of 45–49 years old. This estimation of an older age is corroborated by the degenerative changes in vertebral column. However, the dental findings do not support such an older age range, since only slight dental wear is present, and the parostatus is unremarkable. The age of SM6 cannot be further specified since the bones and teeth are too fragmented.

### Dentition and nutrition

The Saltmen show few caries, likely due to a low cariogenic diet. According to the archaeobotanic investigations, this is particularly surprising for the Sassanian mining period for which there is ample evidence of horticultural activities and the presence of fresh fruits. Additionally, the miners’ diet can be reconstructed via contextual findings of animal bones and macrobotanical remains as well as of paleofaeces that provide the best insight into food consumption [N. Boenke in [4], 55–74]. According to the macrobotanical remains discovered at the excavation, which were processed by wet sieving, there were remarkable differences between the Achaemenid and Sassanian periods. While the grain and fruit supply was rather limited in the Achaemenid period (including different variants of barley, some apricots and peaches), the findings dating to the Sassanian is more variable (i.e. barley, wheat, durum and bread wheat were consumed in addition to a high variability of nuts, olives and fruits, such as e.g. grapes, figs, watermelons and plums). Varying degrees of dental wear are present in these mummies. While the Sassanian Salt Men #1 and #2 show heavy dental wear, the Achaemenid Salt Men #4, #5, and also the Sassanian SM6 show only mild wear. For SM4, the low degree of dental wear is consistent with a younger age, while it is not explainable for SM5 or SM6. For the older-aged individuals, especially of SM5, more dental wear would be expected. Although the number of individuals is too low for a meaningful comparison, we may suspect a different way of provisioning and preparing food. While the Achaemenid miners may have taken their provisions to the mine and possibly cooked a stew of meat and barley (similar to other traditional stews previously mentioned by Herodotus), this may have been different in the Sassanian period, when horticultures were in the surrounding regions and supplied the miners with fruits and vegetables. The grain was likely ground or even came to the site ground beforehand which would have added abrasive minerals such as quartz particles to the meal. The more frequent consumption of fresh, often acidic fruits may have further aggravated the abrasion by the added erosive component [25]. The reason why the Sassanian SM6 only shows slight dental wear may be because he is of a possible foreign origin, which was hypothesized with molecular analyses [C. Warinner, A. Bowman in [4], p- 96].

## Conclusion

In conclusion, the ancient Iranian Saltmen are the earliest known and only preserved cases of natural salt mummies. They are a very rare example of historic salt miners, who most likely died in accidental mine collapses. The individuals show a very heterogeneous state of preservation, which mainly results from the varying surrounding conditions in the salt mine. Multiple traumatic lesions have been observed, but no intra-vitam pathologies.

### Study limitations

We admit some study limitations such as incomplete skeletal material and technical limitations (e.g. the CT-scan of SM6 is not sufficient for the analysis of dentition). For histological analyses, only a few small samples were prepared for analysis in order to preserve the integrity of the Saltmen. More invasive procedures would be needed to assess tissue or organ pathologies, which is not appropriate with such valuable human remains.
